# Differential BroadBand (1–16 GHz) MMIC GaAs mHEMT Low-Noise Amplifier for Radio Astronomy Applications and Sensing

**DOI:** 10.3390/s24103141

**Published:** 2024-05-15

**Authors:** Jose Luis Jimenez-Martin, Vicente Gonzalez-Posadas, Angel Parra-Cerrada, David Espinosa-Adams, Daniel Segovia-Vargas, Wilmar Hernandez

**Affiliations:** 1 Departamento de Ingeniería Audiovisual y Comunicaciones, Universidad Politecnica de Madrid, C/Nicolas Tesla, 28031 Madrid, Spain; joseluis.jimenez@upm.es (J.L.J.-M.); vicente.gonzalez@upm.es (V.G.-P.); angel.parra@upm.es (A.P.-C.); 2 Departamento de Teoría de la Señal, Escuela Politecnica Superior, Universidad Carlos III de Madrid, Campus Leganés, 28911 Madrid, Spain; despinosaa@indra.es (D.E.-A.); dani@tsc.uc3m.es (D.S.-V.); 3 Carrera de Ingeniera Electronica y Automatizacion, Facultad de Ingenieria y Ciencias Aplicadas, Universidad de Las Americas, Quito 170124, Ecuador

**Keywords:** monolithic microwave integrated circuit, broadband gallium arsenide, noise figure, radio astronomy, differential low-noise amplifier, stability analysis, figure of merit

## Abstract

A broadband differential-MMIC low-noise amplifier (DLNA) using metamorphic high-electron-mobility transistors of 70 nm in Gallium Arsenide (70 nm GaAs mHEMT technology) is presented. The design and results of the performance measurements of the DLNA in the frequency band from 1 to 16 GHz are shown, with a high dynamic range, and a noise figure (NF) below 1.3 dB is obtained. In this work, two low-noise amplifiers (LNAs) were designed and manufactured in the OMMIC foundry: a dual LNA, which we call balanced, and a differential LNA, which we call DLNA. However, the paper focuses primarily on DLNA because of its differential architecture. Both use a 70 nm GaAs mHEMT space-qualified technology with a cutoff frequency of 300 GHz. With a low power bias $V_{bias}/I_{bias}$ (5 V/40.5 mA), NF < 1.07 dB “on wafer” was achieved, from 2 to 16 GHz; while with the measurements made “on jig”, NF = 1.1 dB, from 1 to 10 GHz. Furthermore, it was obtained that NF < 1.5 dB, from 1 to 16 GHz, with a figure of merit equal to 145.5 GHz/mW. Finally, with the proposed topology, several LNAs were designed and manufactured, both in the OMMIC process and in other foundries with other processes, such as UMS. The experimental results showed that the NF of the DLNA MMIC with multioctave bandwidth that was built in the frequency range of the L-, S-, C-, and X-bands was satisfactory.

## 1. Introduction

Radio astronomy and sensing applications need to use very high-sensitivity receivers to be able to detect extremely weak signals from deep space. In addition, this high sensitivity is essential in electromedicine and, to a lesser extent, in certain applications in the industrial sector. It is notable how wireless sensor networks (WSN) have promoted great attention due to their versatility and uses in various sectors, such as healthcare, military, industrial automation, and urban intelligence [1,2,3,4,5,6]. In all these cases, receivers must present extremely low figures of merit, which implies using the most innovative technologies to achieve that objective [7,8,9,10,11]. High-electron-mobility transistors (HEMT), made of Gallium Arsenide (GaAs) or Indium Phosphide (InP), seem to be the most suitable candidates for obtaining low noise. The best performance in low-noise amplifiers (LNAs) has been achieved with InP technology [12,13,14,15]. However, these have several drawbacks. For example: (1) the high manufacturing cost, (2) the low breakdown voltage, (3) the greater fragility, and (4) the lower stability compared to pure GaAs technology. This has led to hybrid technologies that make use of InP channels on GaAs wafers. Furthermore, these hybrid technologies have given rise to pseudomorphic transistors (pHEMT) and metamorphic transistors (mHEMT). In recent years, excellent results have been obtained in LNAs manufactured on metamorphic structures [15,16,17,18,19].

Recent major radio astronomical developments, such as the SKA (Square Kilometer Array) and the VLBI2010 (Very Long Baseline Interferometry), have used balanced broadband antenna feeders. The use of very dense arrays in the SKA project, based on a dual-polarization tapered slot antennas (TSAs) array [20], allowed the use of mutual coupling to improve the radiation characteristics of the array. The wise use of this mutual coupling allows for increasing the directive properties of the array, absorbing the impedance variations of the antenna by the connected LNA [21,22]. The work developed in [22,23,24,25,26,27], on the development of dual single-ended and differential structures, has given rise to active balanced feeding networks for the SKA array.

LNAs made with MIC (Microwave Integrated Circuit) technology were traditionally used in the lower frequency bands. In this technology, active and passive components are soldered on the same substrate. This has, among its advantages, the possibility of tuning and adjusting to improve the performance of the amplifier. However, its drawbacks are the difficulty and lack of reliability associated with its assembly.

On the other hand, from an electrical point of view, noise can be reduced using low-loss input-matching networks. However, problems can arise that are associated with instability and the fact that the differential structures necessary for balanced antennas are difficult to implement, especially in broadband devices.

From the point of view of several authors, monolithic MIC (MMIC) technology is appropriate when mass production is required [28,29,30,31]. However, the noise rejection of the LNA is usually worse than that achieved using hybrid technology. But, on the other hand, it turns out to be easier to increase the operating frequency [32].

Therefore, as the main objective for applications such as SKA, VLBI2010, WSN, and medical sensors is to minimize the size, reduce the noise figure (NF), and increase the operating frequency and the corresponding bandwidth, it seems appropriate to work with this technology. This implies that the selected foundries must satisfy all these requirements.

It should be noted that in current sensors and space segments (i.e., mainly in radio astronomical and medical applications), it is necessary both to use antennas and for the antenna-feed networks themselves to be balanced and, possibly, to be active. This means that current baluns seek to have low losses, be broadband, have low noise, and, in many cases, be active [22]. Furthermore, the differential architecture is effective in RF/MW front ends, thanks to the local oscillator (LO) leakage cancellation [26,27,33] that these topologies provide. However, filters at very high frequencies are one of the most difficult components to realize in MMIC when working with differential architectures. Nevertheless, using the differential architecture, the filters can be eliminated, therefore improving the integrity of the front end. Therefore, differential amplifiers (DA) are essential components in differential TRX front ends.

The novelty of this paper lies in the fact that it presents an innovation in the field of RF/MW technology. Specifically, the design of a low-noise differential amplifier with a large bandwidth (DLNA) is presented. In essence, this contribution represents an advance because it offers a satisfying and rare combination of desirable characteristics. For example, a large bandwidth (1–16 GHz), gain greater than 30 dB, common-mode rejection ratio (CMRR) approximately equal to 40 dB, and noise levels approximately equal to 1.0 dB. Here, the noise figure in differential mode measured “on jig” and using the OMMIC foundry is less than 1.4 dB. Several highly regarded designers and manufacturers of commercial DLNAs can be identified, including MACOM (Lowell, MA, USA)^®^, QORVO (Greensboro, NC, USA), MiniCircuits (Brooklyn, NY, USA)^®^, and United Monolithic Semiconductors (UMS) (Villebon-sur-Yvette, France), among others.

To the best of our knowledge, there are currently no commercially available DLNAs on the market that exhibit the same characteristics as those presented in this research. The absence of commercial alternatives serves to highlight the complexity of the challenge we face. In short, designing and implementing a DLNA with high bandwidth and low noise levels is a considerable technical challenge due to the common use of single-ended structures in normal practice. Consequently, the development of a DLNA that can efficiently feed broadband differential antennas could be considered innovative and unconventional.

Finally, in numerous applications, including radio astronomy, the utilization of differential antennas is of paramount importance. These antennas permit the capture of signals from a broad spectrum while simultaneously minimizing noise. Consequently, the integration of a DLNA with the characteristics proposed in this research paves the way for the development of more effective and precise reception and transmission systems.

In this paper, the design and measurement stages are described, and a comparative analysis is performed between the measurements and simulations carried out. A brief description of the DLNA technology and the design process is presented in Section 2. Section 3 describes the analysis process of the design mounted on the carrier and compares it with a single-ended LNA. Furthermore, in Section 3, the total stability analysis of the designed MMIC is conducted, and the setup carried out for the analysis is described. Section 4 shows the measurements made with the device mounted on a support, and the obtained results are compared with both the simulations and the single-ended structure. Finally, the conclusions are given in Section 5.

## 2. Issues of Broadband DLNA Design

### 2.1. Process D007IH

**Figure 1:**
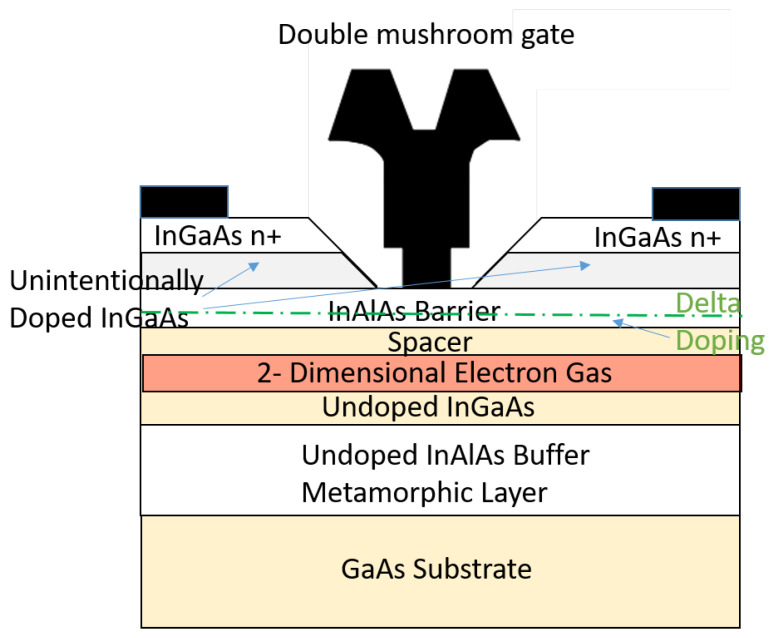
Active-layer profile of D007IH MMIC process.

The D007IH process is a 0.07 μm GaAs on mHEMT technology from OMMIC-MACOM. The first version of mHEMT technology dates back to 2007. Furthermore, this technology is currently fully established and has a Space-Qualified Process (SQP). Some features are as follows:

- The letter *D* means the transistors are in depletion mode and use double-mushroom gates (see Figure 1).
- The number *007* means the transistors are built in an mHEMT technology process with a 70 nm gate length.
- The letters IH mean the active component is InP-doped based on the active layer or employs a high-indium (In)-content epitaxial active layer (it employs InGaAs–InAlAs–InGaAsInAlAs epitaxy with 52%/70% indium content on a metamorphic buffer over a GaAs semi-insulating substrate) (see Figure 1).

The OMMIC process provides a current gain cutoff frequency $f_{T}$ = 300 GHz and a maximum oscillation frequency $f_{max}$ = 350 GHz. The wafer is thinned down to 100 μm. This process is suitable for applications up to 150 GHz with a cutting-edge figure of merits in terms of gain, noise, and reliability. As representative data of the MMIC process at noise level, it should be noted that it has $NF_{min}$ = 0.5 dB at 30 GHz, with a drain-source voltage $V_{DS}$ = 1.3 V. However, the drawback of the process is that the breakdown voltage is very low, $V_{BR}$ = −3 V. Table 1 shows the most important characteristics of the MMIC process, which were provided by the foundry.

Moreover, the MMIC process includes precision Tantalum Nitride resistors (TaN resistors), high values titanium tungstosilicate resistors (TiWSi resistors), Metal–Insulator–Metal capacitors (MIM capacitors), inductors, air-bridges, via-holes through the substrate, and CAD models for passive elements. Microstrip transmission lines are available for the design.

The authors of this article used the OMMIC D007IH process because it has the lowest NF of all the available processes. On the other hand, comparisons with other processes from other open foundries cannot be shown because to have access to the libraries of said processes, a non-disclosure agreement (NDA) must be signed. Moreover, the NDA clearly specifies that neither publications nor public comparisons between processes and foundries can be made.

### 2.2. Transistor Selection and Architecture of the LNA

The first stage in the design of the LNA consisted of selecting the transistor so that it could be adequately characterized. The gate width and the number of fingers of the transistor are included here. Furthermore, to establish a compromise between the required parameters, both the space needed to carry out the construction of the MMIC and the cost per mm^2^ must be taken into account. The required parameters are as follows: noise figure (NF), gain, voltage standing wave ratio (VSWR), stability, and bandwidth. Moreover, in the design presented in this research, the starting requirements were as follows:

1. Gain, *G* > 26 dB in a frequency band ranging from 1 to 16 GHz. The LNA circuit gain in this design was chosen to be at a moderate range of around 26 dB to 33 dB. The upper gain value is to prevent the circuit from oscillating, which commonly happens in very high-gain circuits. Input and output return loss is specified to be better than 5 dB.
2. Noise figure, NF < 1.4 dB. In this MMIC, the NF is targeted to be better than 1.4 dB over a very wide range of frequencies in the band of interest from 1 to 16 GHz.
3. The selected architecture must be differential to minimize the effects of common-mode noise so that it can also function as an active balun in reception antennas or of the sensor with differential power.
4. There are no compression point requirements at the output at 1 dB ($Pout_{1dB}$), but a high level of compression will be positively valued.

**Figure 2:**
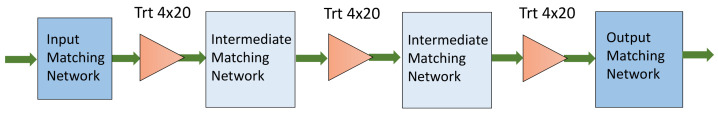
MMIC architecture.

**Figure 3:**
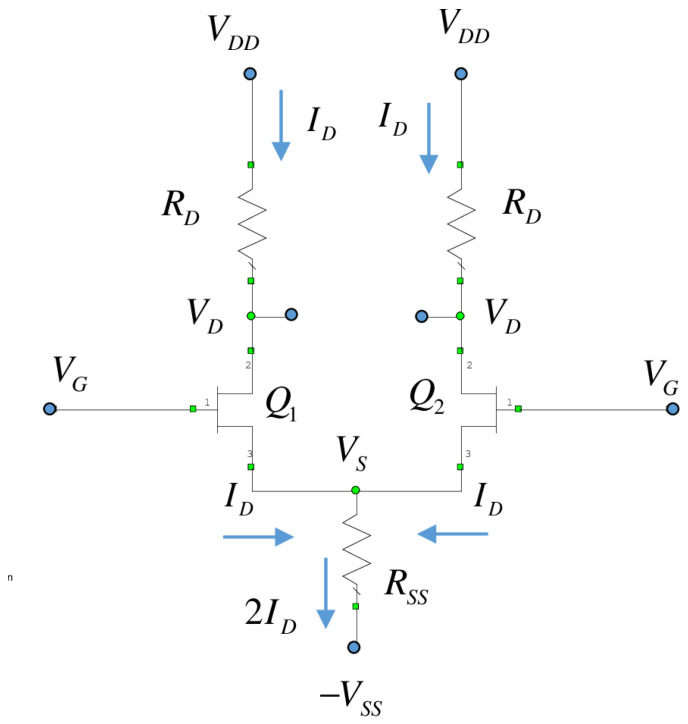
Scheme of the differential stage.

With these gain requirements, a three-stage architecture was selected for the amplifier, as shown in Figure 2. This figure shows that the proposed LNA consists of seven stages: three amplifiers, the input and output matching networks, and two interstage matching networks. In addition, each amplification stage is a differential stage like the one shown in Figure 3. The first stage should have a noise figure (NF) that is as low as possible because this stage contributes the most to the total amount of noise in the overall amplifier, as predicted by the Friis equation [34]. Additionally, the size chosen for the transistors of all the stages of the designed amplifier corresponds to the selected one: 4 fingers × 20 μm gate periphery. Furthermore, a four-fingered MESFET was used to minimize the parasitics in the gate pad due to their parallelism and, at the same time, succeed in an adequate gain suitable for the design specification [35,36].

In general, it has been assumed that a single-stage amplifier configured in single-ended mode has approximately the same gain as a stage in differential mode. In this case, the minimum gain value with this technology (i.e., OMMIC D007IH) is obtained at the highest frequency of the working band (i.e., 16 GHz). Furthermore, the results of the simulation (using the AWR software) indicated that a gain of approximately 11 dB would be required to achieve a minimum noise level. This led to the decision to adopt a three-stage amplifier architecture, as a gain of greater than 26 dB (*G* > 26 dB) was necessary to meet the design objectives.

It is not feasible to select a two-stage amplifier due to the expected gain, which would be approximately 20 dB. This would not reach the minimum gain requirement (*G* > 26 dB), even without considering the losses that would be incurred due to the use of different matching networks.

Conversely, the use of a four-stage amplifier would result in a gain of approximately 40 dB. However, the use of four-stage amplifiers is not common due to the potential for significant design issues, particularly in terms of stability. In particular, in RF (radio frequency) and MW (microwave) designs, high gain is associated with instability due to radiation feedback. This feedback is from unwanted signals that are radiated through the tracks or any metallic element or slit [37]. Therefore, at a practical level, broadband amplifiers with gains close to or greater than 40 dB are not usually manufactured. If these gain values are needed in the transmission or reception chains, several MMICs with interspersed attenuators are usually placed to avoid the risk of unwanted oscillations in RF and MW.

Accordingly, the selected three-stage amplification architecture (see Figure 2) is a configuration that meets the proposed minimum gain requirement of less than 40 dB. This choice allows the gain to be sufficiently high to achieve the design objectives while maintaining the stability of the amplifier. The proposed three-stage architecture has been designed to ensure satisfactory performance in terms of gain and noise.

With respect to Figure 2, to prevent the second and third stages from excessively increasing the total NF of the MMIC, it is necessary that the gain of this first stage be large enough (close to 9 dB). It is important to note that in the first stage, minimizing noise is of primary importance, while in the second and third stages, maximizing the gain is the primary objective. Similarly, the second and third stages contribute a greater gain to the total MMIC than the first stage. The gain of these last two stages is approximately 12 dB, while the NF of these stages is slightly greater than that of the first stage.

Regarding the gain–bandwidth product parameter, it is essential to highlight that, in contrast to low-frequency electronics, the design of RF and MW amplifiers, including both differential and non-differential amplifiers, does not employ this parameter as a design criterion. However, it is utilized as a figure of merit for designs constructed in RF and MW. Additionally, it is employed as a parameter to compare broadband amplifiers in RF and MW. In essence, the performance of broadband LNAs is constrained by the performance of the devices at the highest frequency of the band.

In low-noise applications, the transistor is biased at an operating point that is a compromise between low noise and the maximum gain typically obtained at higher currents [38]. This operating point, at a practical level, is usually a value between 15% and 20% of the maximum value of the drain-to-source current ($I_{DSS}$). In our case, because OMMIC provided us with the noise and gain parameters of its transistors based on polarization, it was decided to select the transistor that had the lowest possible noise and that the gain was acceptable for the proposed design. In this research, the bias point (i.e., drain-to-source voltage ($V_{DS}$), gate-to-source voltage ($V_{GS}$), and drain current ($I_{D}$)) chosen for the OMMIC 4 × 20 gate length mHEMT transistor was $V_{DS}$ = 1.3 V, $V_{GS}$ = −0.6 V, and $I_{D}$ = 19 mA.

Since the circuit is symmetric, the differential/common-mode method was the preferred method to solve this circuit. The equations given by (1) for common mode and (2) for differential mode were used.

(1) $$\frac{v_{oc}}{v_{c}}=-\frac{g_{m}\cdot R_{D}}{1+2g_{m}\cdot R_{SS}+R_{D}/r_{o}}$$

(2) $$\frac{v_{od}}{v_{d}}=-2g_{m}\frac{r_{o}\cdot R_{D}}{r_{o}+R_{D}}.$$

where $v_{c}$ is the common voltage between the gates of the differential pair, $v_{d}$ is the differential voltage between the gates of the differential pair, $v_{oc}$ is the common-mode output voltage, $v_{od}$ is the differential-mode output voltage, $g_{m}$ is the transconductance of the current-dependent generator, $r_{o}$ is the output impedance of the small-signal model of the transistor, $R_{D}$ is the drain resistance, and $R_{SS}$ is the equivalent resistance of the current source.

Regarding the current source, it was synthesized with the help of a transistor, $Q_{S}$, of 4 × 20 μm. Furthermore, with the help of the reference resistor, the source provided a constant current to the differential pair. However, the point where the source is fed is a virtual ground.

### 2.3. DLNA Matching Networks Strategy and Design

One of the critical points in the design of amplifiers is to synthesize the input and output impedance matching networks. In the case of LNAs, the input impedances are defined by the measurements or by the model given by the manufacturer to obtain the minimum noise values. In this work, the OMMIC foundry provided the noise models and the values of the optimal noise impedances to obtain the minimum noise value. In our case, these impedances are very far from the impedance of 50 Ω and are also complicated to realize or synthesize in a band as wide as that required by the proposed design.

The noise factor is given by Fukui’s Equation (3) [39,40].

(3) $$F_{stage}=F_{min}+\frac{G_{N}}{R_{S}}{\left|Z_{S}-Z_{SOPT}\right|}^{2}$$

where $F_{min}$ is the minimum noise factor, $G_{N}$ noise admittance, $R_{S}$ is the real part of complex source impedance, $Z_{S}$ is the impedance of source, $Z_{SOPT}$ is the optimum noise impedance source, and $F_{stage}$ is the noise figure of the amplifier stage. Using optimum noise matching, the minimum achievable noise figure of an LNA ($NF_{min}$) is obtained. On the other hand, power gain (conjugate impedance matching) yields the maximum available power gain for a circuit. Unfortunately, these two matchings are contradictory, and hence, both maximum available gain and minimum noise figure are not simultaneously possible.

In this case, (3) tells us that our minimum noise impedances $Z_{SOPT}$ and maximum gain impedances are usually very far apart. This means that to obtain a good broadband design, a compromise must be reached between both values, sacrificing gain to obtain as small a noise figure as possible. Also, both values must be as close as possible so that the minimum possible noise can be obtained with an acceptable gain.

This problem has been deeply studied, and different techniques have been used to solve it. In this sense, one of the most used techniques has been inductive degeneration [41,42,43,44,45]. In short, inductive degeneration consists of placing an inductance or transmission line with an inductive effect in the source or emitter (in the case of bipolar transistors) of the transistor that allows the optimal impedances of minimum noise to be brought closer to the desired impedance. Furthermore, this must be done while ensuring that the cost of decreasing maximum gain is small. However, on the other hand, the worst drawback of inductive degeneration is the fact that putting inductances or transmission lines in the source or emitter of the transistors substantially increases the instability of the amplifier. Therefore, the risk of LNA oscillation increases. Moreover, the risk of oscillation is greater if the transistor that makes up the LNA has a high gain level. Therefore, taking into account the pros and cons, it is recommended that inductive degeneration be used very carefully, always ensuring that its use does not cause oscillations [41,42].

The architecture of the proposed DLNA is a three-stage structure (see Section 2.2). Furthermore, as predicted by (4), the Friis equation [34], the first stage is the one that contributes the most to the total noise of the MMIC. This is our case. According to [34], the first stage must be optimized for noise performance and subsequent stages for gain boosting. Therefore, in this design, the differential stage is optimized for noise, while the second stage is used to improve the gain.

(4) $$F_{N_{3stage}}=F_{1}+\frac{F_{2}-1}{G_{1}}+\frac{F_{3}}{G_{1}\cdot G_{2}}$$

where $F_{1}$, $F_{2}$, and $F_{3}$ are the noise factors of the first, second, and third stages of the amplifier, respectively, and $G_{1}$ and $G_{2}$ are the gains of the first and second stage of the amplifier, respectively.

It is well known [46,47,48,49] that if the simulation of the final circuit results in a circuit that satisfies the classical stability condition, then the μ factor [47] achieved will be greater than 1 in the amplifier’s operating frequency band. This requirement is necessary but not sufficient because complying with it does not ensure that the circuit is stable, as demonstrated in [48]. Specifically, instabilities could occur in a single stage or in the modes of operation that we have [48,49,50].

To ensure the unconditional stability of the amplifier and guarantee that no instabilities appear, each stage of the amplifier is loaded with an RC network at the output (i.e., padding at the output), substantially avoiding worsening the noise of the total set. This ensures that the multistage amplifier is absolutely stable compared to any hypothetical input or output port. Furthermore, it is ensured that the designed DLNA achieves the gain and NF value required by the design. That said, in the design shown in this research, transmission lines were also added to help, together with the networks, to obtain the optimal loads for the proposed amplifier.

Another important aspect to highlight in the design of the DLNA is that in the first differential stage, an instability appeared that, from the authors’ point of view, could cause the appearance of oscillations. The above situation could occur even by putting the RC padding networks at the output. Therefore, to solve this problem, it was decided that the first stage would have its source connected directly to the ground of the circuit, not to the virtual ground provided by the differential architecture. Regarding the architecture of the second and third stages, and since the common-mode rejection ratio (CMRR) was a fundamental parameter sought in the design, these remained the same as the classic stages of a differential amplifier. This allowed us to guarantee that both the CMRR and the gain were as high as possible. The CMRR parameter of a differential amplifier is the rejection made by the device to unwanted input signals that are common to both input leads relative to the wanted difference signal. An ideal differential amplifier would have infinite CMRR. However, this is not achievable in practice. A high CMRR is required when a differential signal must be amplified in the presence of a possibly large common-mode input. All the above was also done in this research to ensure the total stability of the DLNA and that the NF was as low as possible.

All the factors that have been described previously, and with a view to performing stability simulations, have led the authors of this work to model the transistors of each stage as passive loads. A compromise was established between the noise, stability, and gain of each stage when carrying out the final design of the DLNA. Next, the two phases followed for the final design of the DLNA built in this research are described.

In the first phase, MMIC optimization was carried out at the circuit level. Here, the lines and passive elements were simulated using the models provided by the OMMIC foundry. Furthermore, with the help of the transmission-line model provided by the OMMIC process design kit (PDK), the input and load impedances were matched before using the electromagnetic (EM) simulator (The AXIEM electromagnetic tool from AWR software, version 15), starting from the first stage and ending at the output stage.

**Figure 4:**
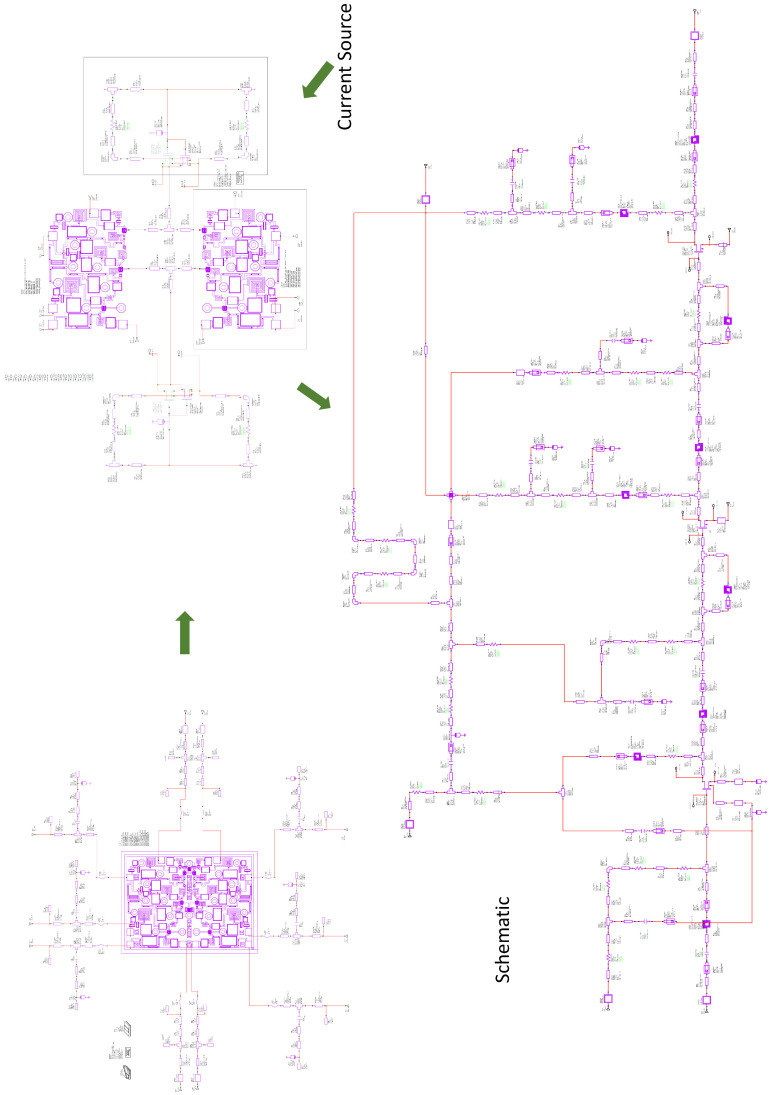
Complete electrical scheme of the full Differential LNA.

**Figure 5:**
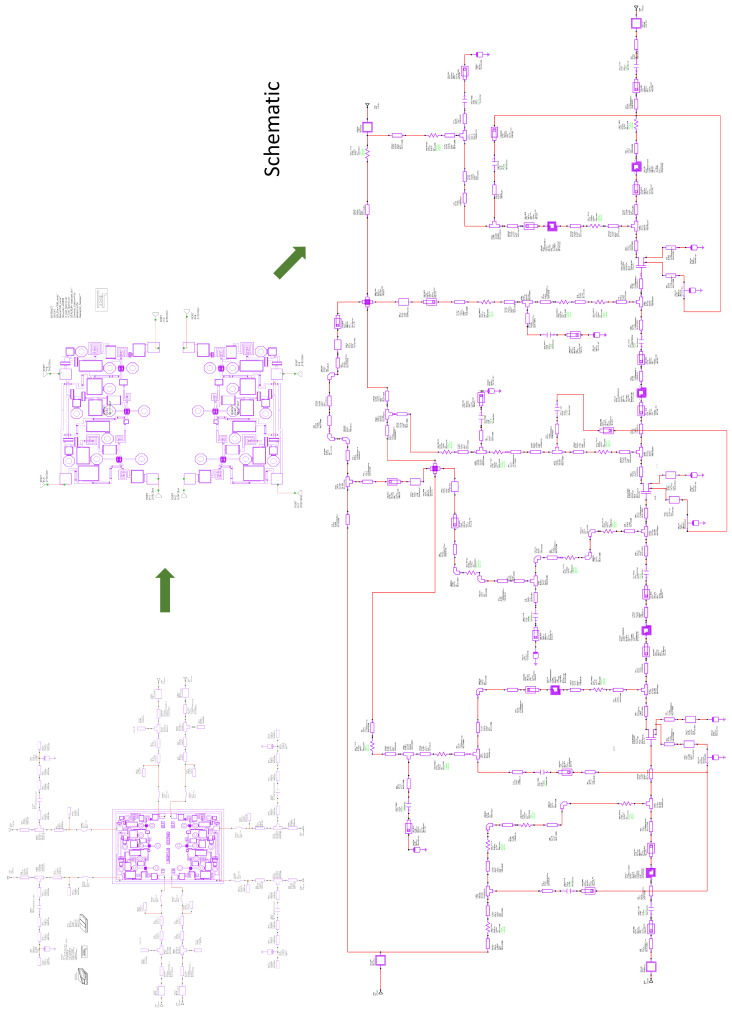
Complete electrical scheme of the Dual LNA.

In the second phase, DLNA adjustment was performed using EM simulation. The EM simulation made it possible to model all the lines and passive elements that make up the MMIC. The complete DLNA was passed to the EM model, and optimization was carried out to maximize gain values and minimize noise. The truth is that this optimization did not turn out to be very complex. This was because the PDK circuit model, obtained in the first phase, and the working frequency band made it possible for the response of the circuit modeled with the PDK to be very close to the response obtained with the DLNA EM model. Figure 4 includes a complete electrical scheme of the full Differential LNA containing component values (resistors, capacitors, transmission lines); the single-ended version of the LNA is shown in Figure 5.

**Figure 6:**
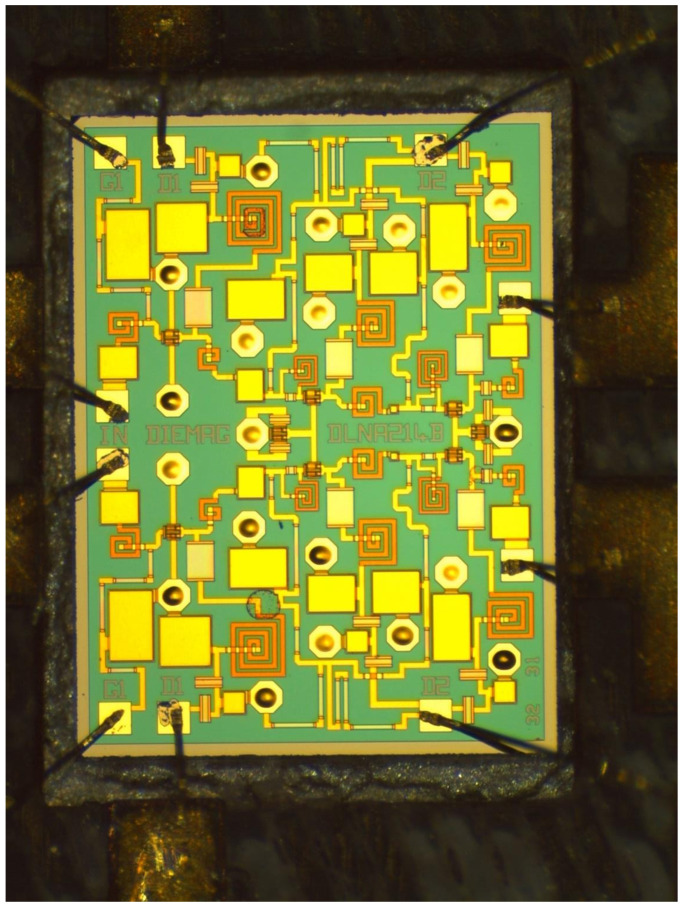
A micrograph of the differential broadband MMIC low-noise amplifier. The size of the chip is 1.5 mm × 2.0 mm.

Taking into account everything stated above, the design of the DLNA MMIC shown in Figure 6 was obtained. Additionally, the opportunity to have a second design on the same shared wafer was taken. Therefore, a second LNA design was shipped on the same shared OMMIC wafer. This second design did not use the differential architecture and served to compare the results of gain, NF, and CMRR in the same process and on the same wafer.

**Figure 7:**
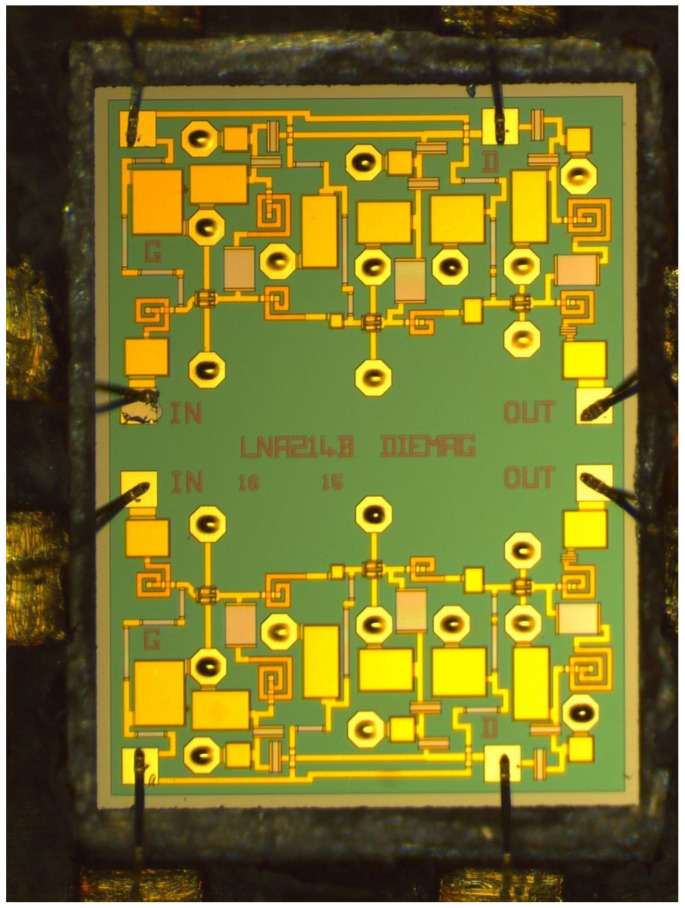
A micrograph of the dual single-ended broadband MMIC low-noise amplifier. The size of the chip is 1.5 mm × 2.0 mm.

Regarding the second design, this is a double single-ended LNA design, i.e., it contains two single-ended LNAs. Furthermore, this MMIC has the same number of stages as the proposed DLNA (i.e., 3 stages). Also, it uses the same type of transistor in each of the stages of the LNAs and has the same bias point. All this was carried out so that this design would have noise and gain characteristics that were similar to the characteristics of the design proposed in this paper. This allowed us to verify that the performance of the DLNA with the differential structure is better than the performance of a single-ended LNA design. Figure 7 shows the design of the double LNA that was built with the same transistors, the same objectives, and the same characteristics as the differential design.

### 2.4. Chip Simulations

**Figure 8:**
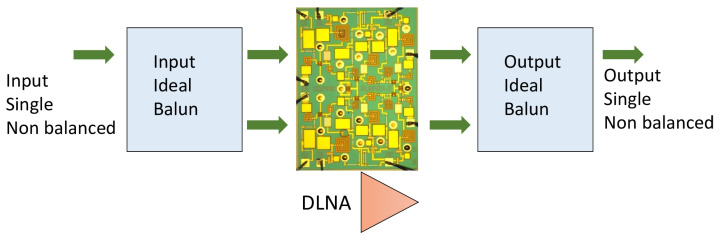
Block diagram that was built to perform the characterization of the MMIC.

To carry out both the simulations and the final optimization of the chip, the block diagram to perform simulations shown in Figure 8 was built. In this test bench, ideal baluns [51,52,53] were used to evaluate the characteristics and performance of the designed chip. These ideal baluns are provided by the AWR simulator through the mixed-mode converter that transforms a common input signal into a differential-mode output. This model is commonly used for differential circuit analysis but can also be used as an ideal power combiner/splitter. The scheme shown in Figure 8 is valid for the characterization of both the small-signal and large-signal parameters that will be carried out in subsequent sections.

The AXIEM electromagnetic tool from AWR software version 15 allowed us to assess the effect that lines and passive elements have on the final behavior of the designed broadband DLNA. In the simulations carried out, the circuit model for the lines and passive elements, provided by the OMMIC foundry, and the electromagnetic model that the AWR software allows modeling were studied.

**Figure 9:**
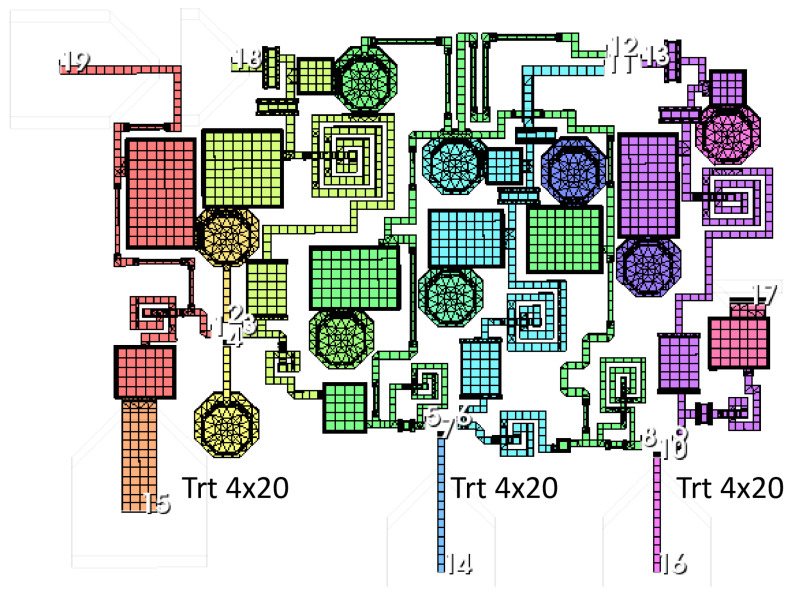
Grid for the electromagnetic simulation of the broadband DLNA MMIC.

**Figure 10:**
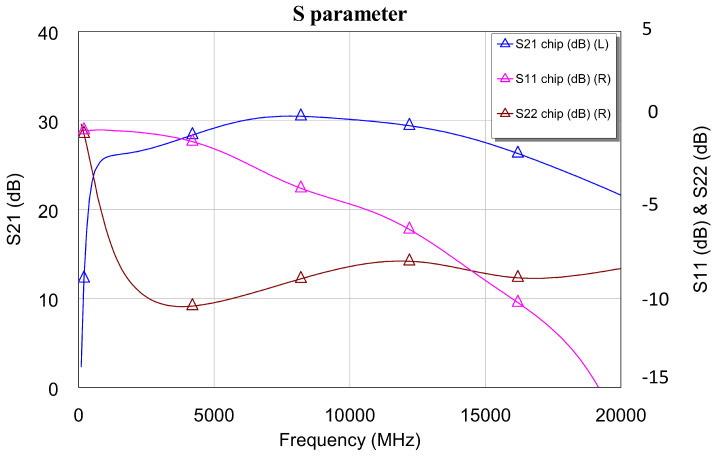
Simulated S parameters of the DLNA MMIC.

**Figure 11:**
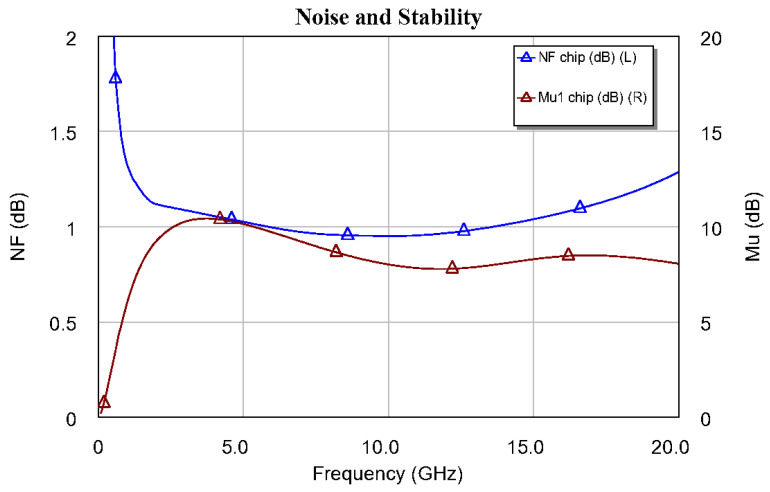
DLNA MMIC noise figure and stability of the electromagnetic simulation.

Figure 9 shows a detail of the meshing of the upper half of the DLNA and the grid for the electromagnetic simulation of the broadband DLNA MMIC designed in this research. As shown in the AWR help, the AXIEM mesher automatically generates a hybrid mesh consisting of mixed triangular and rectangular cells. The mesh is a full surface mesh that can accurately model both thin and thick conductors. AXIEM mesh options can be configured globally by selecting the EM simulator options. This mesh is related to the wavelength at the highest frequency of the LNA because it is the frequency that determines the lower accuracy of the calculation in AXIEM. However, in this research, the default meshing provided by AWR was used. In addition, Figure 10 shows the S parameters of the differential amplifier, and Figure 11 shows the noise figure and stability of the EM simulation. At this point, it is worth mentioning that the circuit simulation served as a good approximation to the final design with the EM simulator. The results shown from the MMIC were simulated with the EM simulator.

In all this, it can be seen that the circuit is fully stable, not only in the operating band but also in broadband. This is due to the good agreement between the electromagnetic simulation and the circuit simulation. This good agreement allowed us to simulate the MMIC networks over short times before carrying out the final simulations that we have shown. Also, it is worth mentioning that the gain value obtained was close to 30 dB and that NF < 1.3 dB at room temperature throughout the bandwidth. Additionally, the output return losses ($S_{22}$) are very low, and the parameter $S_{11}$ has a value that is acceptable for the differential low-noise configuration shown. $S_{11}$ is not matched at low frequencies within the operating range. This is because the bandwidth is extremely large, and what is sought, above all, is output adaptation. This allows DLNA to be used for sensing applications.

Another important aspect to highlight is that to perform the complete analysis of the circuit instability, the EM simulation was carried out across the desired frequency bandwidth. Also, the frequency band increased to consider both much lower frequencies and much higher frequencies. In our case study, we decided to expand the bandwidth to consider from 10 MHz to 200 GHz, taking into account the specifications of OMMIC transistors with $f_{T}$ > 100 GHz.

**Figure 12:**
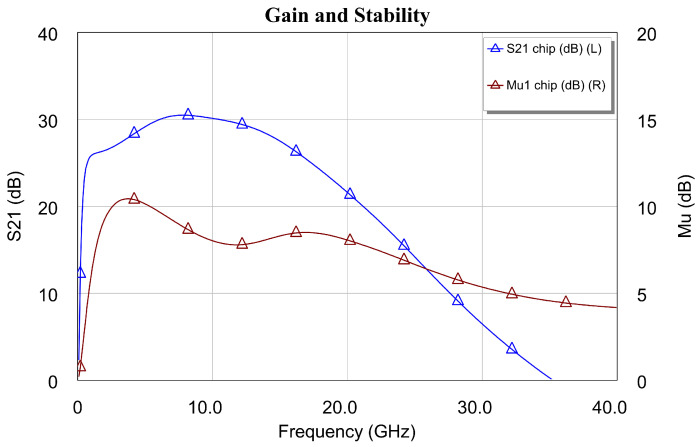
Gain and stability of DLNA MMIC in a broadband analysis.

The stability factor μ [47] is shown in Figure 11 and Figure 12 along with the noise figure (see the left axis of Figure 11) and gain (*G* = $|S_{21}{|}^{2}$, see the left axis of Figure 12). In these figures (i.e., Figure 11 and Figure 12), the stability parameter is greater than 1 (0 dB), and frequency analysis was carried out in the DC-20 GHz and DC-40 GHz bands, respectively. The above was carried out to obtain a clear picture of the gain and NF performance of the designed MMIC. However, although the figure is not shown, the stability factor in the DC-220 GHz band of the DLNA “on wafer” was also analyzed. Here, it was confirmed that the DLNA was fully stable throughout the band, where the design carried out could present some instability. This shows that the amplifier is unconditionally stable at all operating frequencies. Therefore, we can say that a DLNA has been designed to meet all the required features.

As mentioned in Section 2.4, in this work, the circuit in Figure 7 was also designed and manufactured. This circuit is a double LNA with the same noise performance as the DLNA shown. In order not to deviate from the fundamental objective of this paper, which is to design the broadband DLNA, we prefer not to show the results of the stability, NF, and gain (*G* = $|S_{21}{|}^{2}$) simulations of the single-ended design. However, it is worth mentioning that these results are very similar to the results obtained with the DLNA presented.

### 2.5. DLNA Design Procedure Summary

1. Defining specifications and requirements: The first step is to clearly define the DLNA specifications and requirements. This includes determining the operating frequency range (1–16 GHz), the desired gain level (30 dB), the maximum allowable noise figure (<1.5 dB), and other relevant parameters. Establishing these specifications provides a guideline for designing and evaluating the performance of the LNA.
2. Selection of the foundry and the transistor: The OMMIC foundry was chosen due to its current status as having the lowest noise figure among open foundries. The selection of the appropriate transistor is critical to the LNA performance. A transistor is selected based on its ability to meet the design specifications, such as low noise figure and high gain, which are crucial. The OMMIC PDK utilizes available transistor models and selects the polarization point.
3. Design of the RF circuit: After selecting the transistor, the next step is to design the RF circuit of the LNA. This involves determining the topology of the circuit (differential topology) and the number of stages (three stages), as well as arranging components such as inductors, capacitors, and resistors to optimize performance in terms of gain, noise figure, stability, and bandwidth.
4. Initial simulation: After designing the RF circuit, specialized RF circuit design software, such as AWR, is used to perform an initial simulation. This stage allows for the identification of potential design issues and preliminary adjustments to improve DLNA performance.
5. Design optimization: The DLNA design is optimized based on the initial simulation results. This involves adjusting circuit parameters and performing design iterations to improve performance according to previously established specifications.
6. Detailed simulation based on electromagnetic (EM) simulation: After completing the design optimization, a detailed electromagnetic (EM) simulation is performed using the AWR AXIEM RF circuit simulation software. This simulation is crucial in evaluating the performance of the DLNA and ensuring its compliance with the design specifications.
7. Construction and testing: Once the design has been optimized and validated through simulation, the graphic file of the layers and integrated components of the DLNA prototype design (i.e., GDS file) is sent to the foundry for construction of the MMICs. The foundry, whose processes are protected by industrial secrecy, carries out the construction and first experimental tests, as well as visual inspections to verify that the design has been correctly constructed according to the GDS file provided.
8. Measurement, final validation, and refinement: The DLNA undergoes a thorough validation to ensure it meets all initial specifications and requirements. This involves a measurement campaign on the different prototypes to validate that the initial design specifications are met. Once completed, the DLNA is ready for implementation in practical applications.

The proposed LNAs demonstrate a notable improvement in bandwidth through the implementation of various design and optimization techniques and strategies. The strategies employed can be summarized as follows:

1. Proper selection of active devices (transistors): The use of active devices, such as high-frequency transistors, with high bandwidth characteristics (i.e., large $f_{max}$) helps extend the frequency range over which the LNA can operate effectively. In this case, the selected OMMIC foundry process is essential for both noise and $f_{max}$.
2. Broadband impedance matching network design: Implementing matching networks at the input, output, and intermediate stages of the LNA that are designed to operate over a wide range of frequencies improves the frequency response of the amplifier over the entire spectrum of interest.
3. Design optimization: The objective of design optimization is to minimize losses and reflections in transmission lines and connections between circuit components. This is carried out to help maintain the response within the bandwidth and to minimize signal degradation at higher frequencies.
4. Proper selection of MMIC components: The selection of MMIC components is crucial for significantly improving the bandwidth of the LNA. The OMMIC process technology employed exerts a profound influence on the outcome. Therefore, components with a high-quality factor (Q) and commendable performance at high frequencies are utilized. To attain this objective, the inductors employed are minimized. Additionally, capacitors and resistors must be designed to perform optimally over a wide frequency range and with the highest Q possible.

In conclusion, the improvement of bandwidth in LNA design necessitates a comprehensive approach encompassing several factors. Primarily, the circuit design, the selection of the foundry, and the process are addressed. Second, to ensure optimal performance over a wide range of frequencies, both the elements that comprise the MMIC and parameter optimization are considered.

## 3. Simulations Carried Out in the Carrier Circuit

Once what was proposed in the previous sections was carried out, the proposed DLNA and the double MMIC LNA were measured. The measurements made will be shown in the next section. However, the MMICs designed in this research could not be measured “on wafer” because OMMIC did not have this option at that time. Therefore, the measurements of the MMICs designed had to be carried out with them mounted on a board “on jig”, which is mounted on a carrier. This meant that the simulations presented would have to be redone for the board-mounted MMIC.

To do this, the two MMICs were mounted on a board specifically designed to be able to measure these circuits. This board was built on a Cuclad 217 substrate ($\epsilon_{r}$ = 2.2, h = 5 mil) from Rogers Corporation. The input and output connectors that were chosen were ELF-KN 2.92 mm Edge Launch from Southwest Microwave.

**Figure 13:**
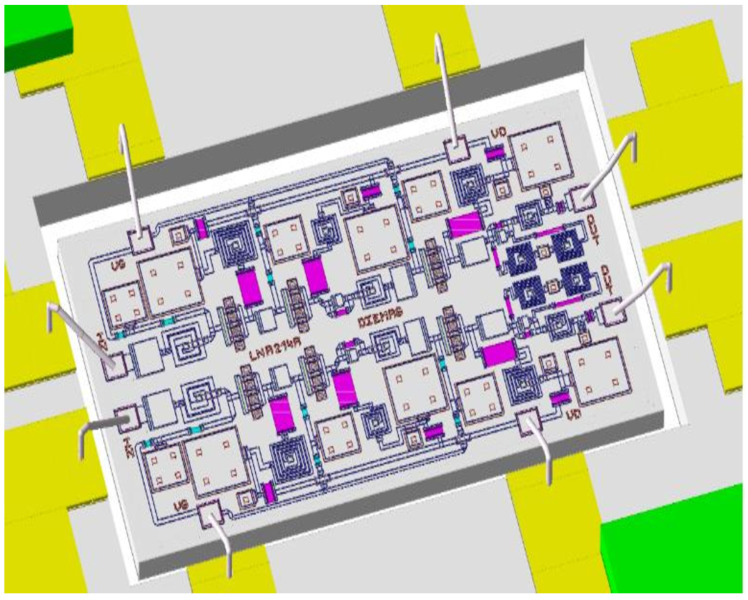
Detail of bonding wires MMIC assembly in the fabricated board to make the measurements (chip size is 1.5 mm × 2 mm).

Once the circuit was mounted on the board, the interconnections between the MMIC and the board were made using the wire bonding method. Figure 13 shows a detail, on a double circuit, of the final arrangement of the joints connected to both the power supply and the input and output of the circuit. The bonding wires must be both circuital characterized and electromagnetically characterized to obtain the true behavior of the MMIC mounted on the carrier and the board.

The bonding wires are close to 150 μm in length at the inputs and outputs, with a diameter of 25.4 μm. The distance between the output/input line of the chip and the board is 70 μm. In this research, the BWIRES models provided by AWR have been used to simulate the connections from the PCB to the MMIC. These bonding wires are formed by a series of segments that compose a bonding wire. This is approximated by three or four linear segments and the parameters defining the orientation of the segments in compliance with standard EIA/JEDEC Standard No.59. This characterization was performed using the 3D EM (Analyst) simulator of AWR (Cadence^®^ AWR^®^ Analyst™ (Cadence Design Systems Inc, San Jose, CA, USA) 3D finite-element method (FEM) electromagnetic (EM) simulation and analysis simulation), version 15.

**Figure 14:**
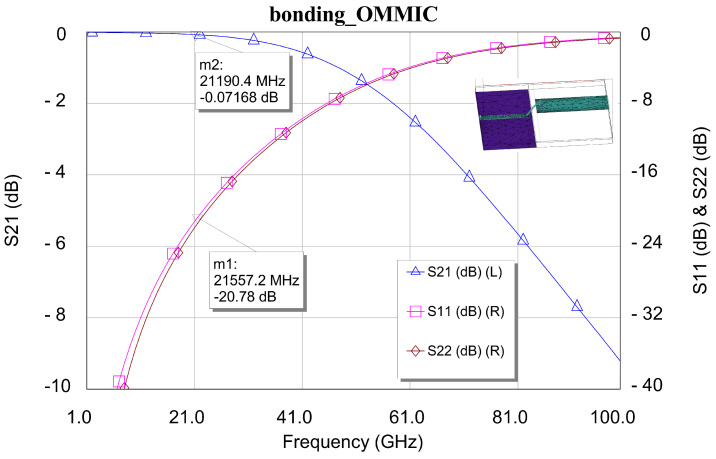
Simulated performance of input and output bonding wires connection.

Next, the circuit was characterized and optimized. Figure 14 shows the results obtained. These results characterize the input and output connections of the MMIC (bonding wires). It can be seen that the results are satisfactory, both for the insertion losses and for the input and output reflections of radio frequency (RF) and microwave (MW) signals. This good performance is due to the quality of the optimization that was carried out so as not to increase or penalize the performance of the LNAs that were mounted “on jig”.

**Figure 15:**
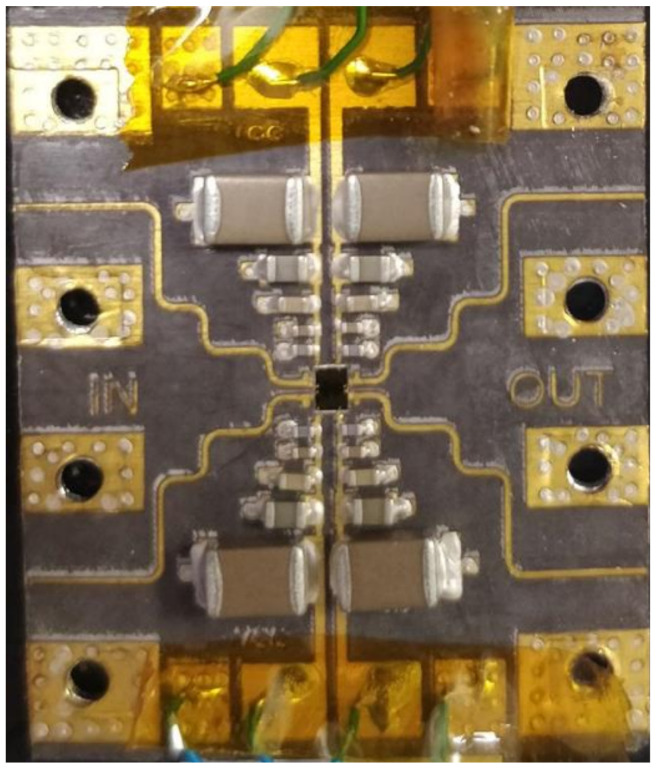
Picture of carrier assembly for DLNA measurement. PCB size is 2.4 × 3.0 ${\mathrm{cm}}^{2}$.

A photograph of the DLNA circuit is shown in Figure 15. The size of the printed circuit board (PCB) is 2.9 × 3.0 cm^2^. Additionally, it is important to point out that the size of the MMIC is 1.5 × 2 mm^2^.

Once the simulations in which the connections of the MMIC to the PCB were carried out, more simulations were performed to find out what the effects of the integration of the designed MMIC with the printed circuit of the board that was designed to be able to carry out the measurements (“on jig”). The above allowed us to evaluate the real performance of the MMIC circuit.

## 4. Measurements

In this research, the measurements were performed by INDRA company, Madrid, Spain, and the comparison between the measurements and the simulations carried out is shown below.

### 4.1. Small-Signal Measurements

**Figure 16:**
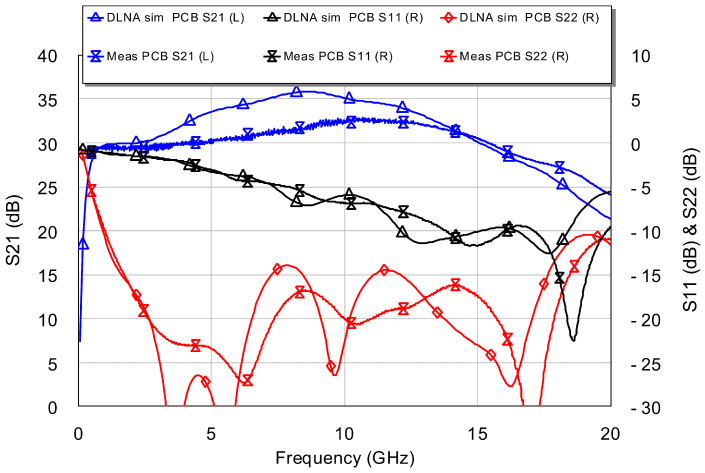
Measurement of S parameters: Comparison between simulation and DLNA measurement.

First, small-signal characteristics were evaluated by measuring S parameters using the Keysight PNA-X vector network analyzer. Electronic calibration was used. The S parameters that were measured are shown in Figure 16. The desired bandwidth and a high gain were obtained, G = $|S_{21}{|}^{2}$. Furthermore, the value of $S_{21}$ is within 30.5 ± 2 dB in the desired band (i.e., 1–16 GHz), with peak value ${\left|S_{21}\right|}^{2}$ = 32.5 dB at 10 GHz. The output matching is satisfactory, ${\left|S_{22}\right|}^{2}$ < −12 dB, and the measurements corroborate the good approximation that exists between the simulations and the measurements.

**Figure 17:**
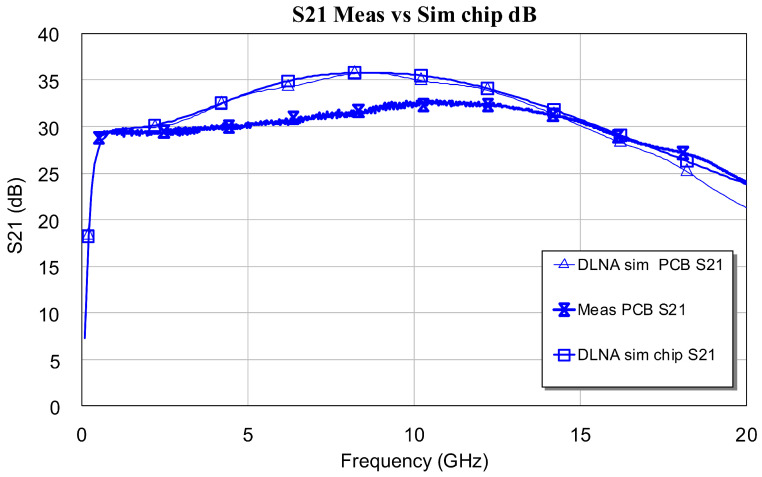
Gain detail: Comparison between simulation and DLNA measurement.

At this point, it is important to mention that there is a small difference between the true gain and the gain estimated in the EM simulation (see Figure 17). In this case, the true gain is greater than the estimated one. The difference is 2.5 dB. The authors of this work think that this difference is due to the fact that EM simulation penalizes losses at frequencies greater than 10 GHz.

At microwave (MW) frequencies, typically between 1 and 100 GHz, it is well known to work with transmission-line techniques and structures referenced to single-ended ports, such as coaxial ports. In addition, differential architectures are not normally used in these frequency bands. Because of this, almost all designs respond to common-mode architectures, or what we call single-ended. This dilemma is currently changing rapidly because communications systems are becoming more complex every day, and it is necessary to use differential architectures. Also, the rapid evolution of MMICs on silicon has helped greatly because this has made it possible to implement these differential architectures with reduced sizes and adequate performance for many of the systems proposed for applications in communications, radar, etc. However, on the other hand, MMICs on silicon do not have as low a noise level as MMICs on GaAs or GaN [54]. Furthermore, the output power of MMICs on silicon is lower than that of MMICs on GaAs or GaN. In this sense, the design presented in this paper is a contribution in the line of carrying out differential architectures with GaAs or GaN technology by presenting a differential amplifier with an operating band from 1 to 16 GHz. This indicates that this design is really very complicated at these frequencies, with this band, and with the use of GaAs technology.

To a large extent, the complication mentioned above is caused by using current sources in the differentials. Current sources make DLNA designs unstable in the MW band. Generally speaking, this is due to parasitic effects and other feedback introduced by the design and current sources mentioned above in GaAs or GaN technologies. In most DLNA designs on GaAs or GaN, these instabilities cause these designs to oscillate. Furthermore, this instability presented by MMICs in GaAs or GaN is not weakly manifested in the designs in MMICs on silicon. This is because these designs have fewer parasitic effects and feedback due to their size and technology. All this means that the majority of DLNA MMICs designed are MMICs on silicon.

This means that high-performance receiver architectures that use differential structures usually must be made with Silicon MMIC or that alternative solutions must be sought using single-ended architectures. For example, if these were needed in differential amplifier architecture, then these amplifiers would be realized using 180-degree phase-shift networks and single-ended architectures. In this work, an ultra-wideband differential amplifier from 1 to 16 GHz is presented, with a noise behavior similar to what the amplifier would have if it were single-ended. Furthermore, the proposed amplifier presents an important improvement in the fact that it has CMRR, which is associated with its differential character. Ideally, a differential amplifier completely suppresses or rejects common-mode signals. Common-mode signals should not appear at the output of the circuit. In short, if the differential amplifier is well designed, at a practical level, this implies that this common-mode gain is very small for some frequencies close to the minimum value that can be measured or the noise of the network analyzer. That is why in the figure of the common-mode rejection ratio (CMRR) measurement, there are CMRR peaks unlike the simulated one because it has not been possible to measure exactly the value of common-mode gain or it is very close either to zero or to the noise of the network analyzer. This is what happens in the CMRR measurement shown in the band from 1 to 4 GHz.

**Figure 18:**
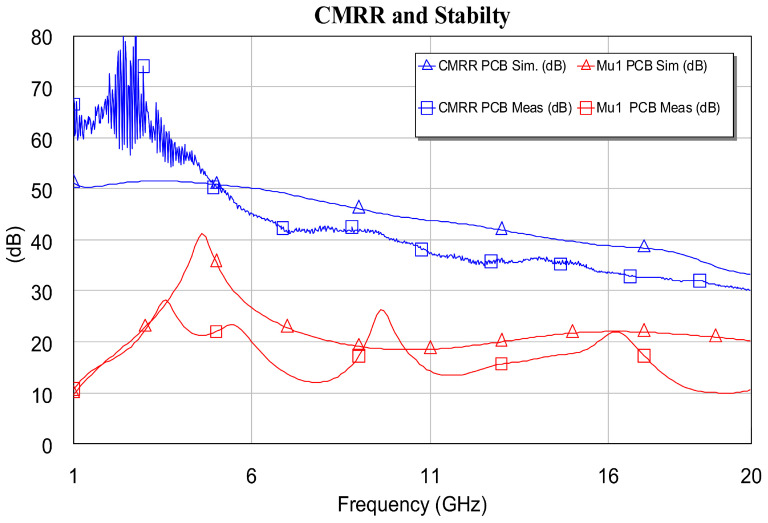
Stability and CMRR measurement: Comparison between simulation and DLNA measurement.

Differential amplifiers are characterized by the large CMRR, a property that non-differential structures do not have. In this sense, to verify the good CMRR value that our DLNA has, the MMIC circuit was measured in common mode. Figure 18 shows the CMRR obtained from the measurements made. Additionally, this figure shows the stability of the proposed DLNA. Stability measurements and calculated stability are shown here. On the other hand, it is worth mentioning that the measured CMRR parameter shows several peaks of high value at low frequencies. Regarding this, the authors of this work think that the above is due to the fact that the DLNA gain in common mode could not be distinguished from noise at low frequencies, and this fluctuation appears in the measurement. Finally, the differences between simulations and measurements are also due to the models provided by the foundries and characterization errors in the EM simulation. However, to understand the reason for the differences, a Monte Carlo simulation analysis of the global circuit was carried out in this research. This allowed us to visualize the sensitivity analysis (yield) of both the designs and the robustness of the designs.

**Figure 19:**
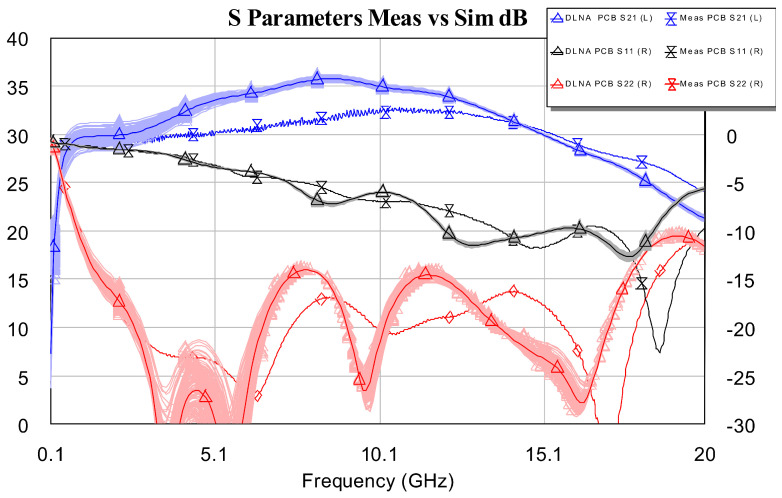
Obtained S parameters using the Monte Carlo simulations of the overall MMIC and the PCB structure.

At this point, it is worth mentioning that manufacturing the MMIC is not an exact process. Therefore, the OMMIC foundry describes the tolerances. Therefore, to characterize the MMIC, design yield analysis was performed with a sample space of 100 simulations using the Monte Carlo method with the PDK provided. In this case, the values of the transistor model provided by the OMMIC foundry were not modified. Therefore, the transistor parameters were kept fixed. However, the values of the tracks and passive components were modified according to the normal distribution. The Monte Carlo simulation result is shown in Figure 19. It can be said that the performance of the MMIC DLNA is satisfactory because the variations are in accordance with the sensitivity analysis carried out.

With respect to the MMIC chip, both in its DLNA version and in its LNA “on wafer” version, it can be said that its performance is also in accordance with the simulations shown in Section 2.4. Therefore, the authors of this work think that it is not worth presenting the results of the “on wafer” chip because these results could be extrapolated by de-embedding the additional structures introduced for measurement.

### 4.2. Noise Measurements

**Figure 20:**
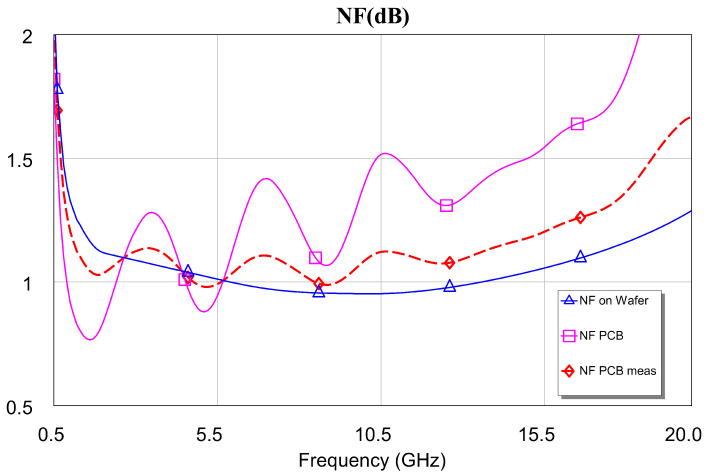
Noise figure measurement: “on jig” measurement of the designed DLNA vs “on jig” and “on wafer” simulations.

Figure 20 shows the measurements made of the final noise figure for ambient temperature conditions. The N8975B noise figure analyzer was used to carry out the noise figure measurements, and the measurements were performed in compliance with [55,56,57]. In the measurements of the DLNA structure, the simulations were introduced (“on jig”). As a result, it was obtained that the value of the “on jig” noise factor was lower than expected and higher than the value of the “on wafer” noise factor, as would be expected [58,59]. As we can see, there is a very good agreement between the simulations and the measurements carried out. NF < 1.3 dB throughout the frequency band of interest (i.e., 1–16 GHz). This value is slightly higher than the value obtained from the “on wafer” MMIC simulation due to the use of a printed circuit made up of tracks and capacitors to facilitate measurement. If the de-embedding discussed in Section 4.1 were performed, then it would be observed that the NF would be below the simulated value for the “on wafer” case.

As one of the fundamental applications of MMICs is in the field of radio astronomy, we think that it is necessary to evaluate the noise temperature of these circuits to be able to consider whether the performance of the amplifier under cryogenic conditions will be satisfactory [60,61,62,63,64,65,66,67,68].

Although it is well known that the noise factor suffers an appreciable reduction depending on the temperature [67], normally, to characterize the noise behavior of cryogenic LNAs, the noise temperature is usually measured [68,69,70] without using the noise factor. That said, if noise temperature measurements are not performed, then the study of noise temperature and noise factor of LNAs with temperature requires complex calculations [71]. To this end, some works, such as [71], even resort to quantum mechanics [72] to analyze the behavior of the noise factor at cryogenic temperatures. In this research, no cryostat was used. In addition, the measurements carried out were made at room temperature (i.e., approximately 298 K). Furthermore, according to [71], the cryostat temperature range to reduce the noise factor by 10% to 15% is approximately 200 K. This decrease occurs in the noise factor (rational number), and is due to a lower mobility of the particles as a function of the thermal decrease [67].

**Figure 21:**
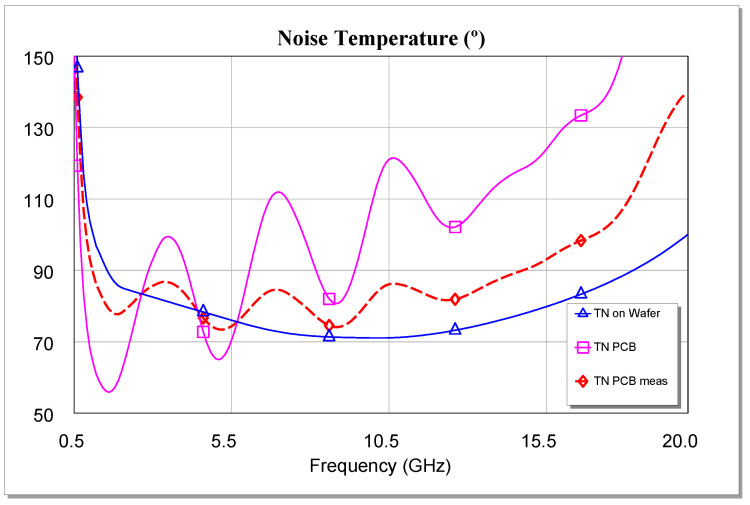
Simulation and “on jig” measurement of the amplifier noise temperature (with $T_{o}$ = 290 K).

Figure 21 shows the temperatures obtained, both from the measurements and the simulations carried out for the temperature $T_{o}$ = 290 K. We can conclude that both the gain and the noise temperature show satisfactory performance throughout the entire bandwidth of the designed DLNA, showing better than expected values.

### 4.3. Large-Signal Measurements

**Figure 22:**
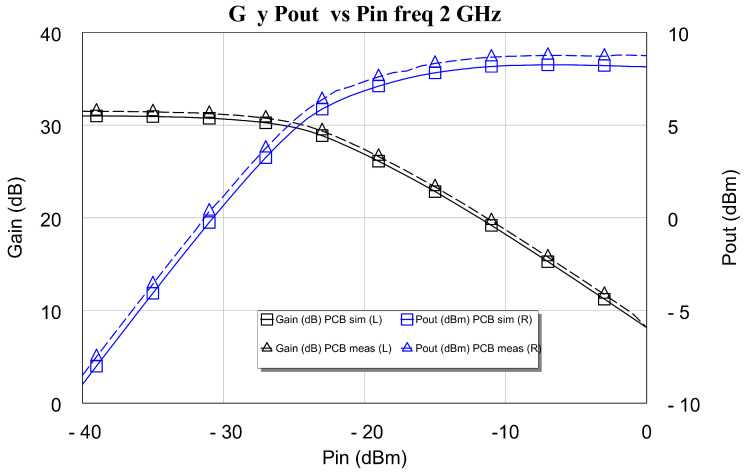
Simulation and measurement of power output at 1 dB gain compression point, $P_{out1dB}$, at 2 GHz.

**Figure 23:**
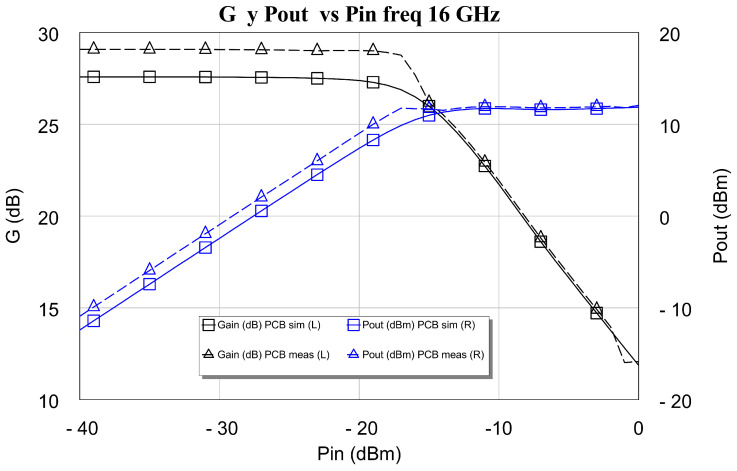
Simulation and measurement of power output at 1 dB gain compression point, $P_{out1dB}$, at 16 GHz.

In the design of LNAs, the priority is always to obtain the lowest possible noise figures. However, we think it is appropriate to build a small, large-signal model of the designed LNA and carry out modeling of the behavior of its compression point. To do this, both the simulation and the measurement of the gain *G* and the output power $P_{out}$ as a function of the input power $P_{in}$ were carried out (see Figure 22 and Figure 23). The measurement tests were carried out with a vector network analyzer (VNA). This instrument was the four-port Keysight PNA-X 5224B. Furthermore, as LNAs generally have a compression point that is lower than the maximum output power provided by this network analyzer (13 dBm), in this research, it was not necessary to use external amplifiers to measure the compression point of the DLNAs. Also, the test bench used is the standard one, with the device under test (DUT) (i.e., the LNAs) located between the measurement ports. Moreover, the power-sweep range must be large enough to drive the amplifier under test from its linear region of operation to its region of compression. Modern network analyzers typically provide power sweeps with more than 30 dB of range, which is enough to drive most amplifiers into compression. It is also especially important to sufficiently attenuate the output of high-power amplifiers not only to prevent damage to the receiver of the network analyzer but also to keep power levels low enough to avoid receiver compression. These two plots contain simulations and tested values of power output at 1 dB gain compression point, $P_{out1dB}$, both at 2 GHz and 16 GHz.

The results of the measurements and the simulations carried out are very similar at the frequencies of the lower band, as is the case of 2 GHz. This is because the measured gain value and the compression point are slightly above the simulation (approx. 0.5 dB). At the higher frequency, 16 GHz, the small-signal gain is of the order of 2.5 dB greater than the simulation of the measurement carried out. This greater gain obtained in the measurement causes the differential amplifier (DLNA) to compress earlier in the measurement than in the simulation, as shown in Figure 22 and Figure 23. In these figures, it is observed that the compression point at the output, $P_{out1dB}$, obtained in the measurement, especially the one shown in Figure 22, is greater than that provided by the simulation. Therefore, it can be concluded that our compression point at 1 dB, $P_{out1dB}$, is always greater than 8.5 dBm throughout the band, with a maximum value equal to 12 dBm at 10 GHz.

### 4.4. Single-Ended vs. Differential LNA

To take into account the overall performance of the DLNA MMIC presented in this research, we think it is important to take into account the figure of merit (FOM). In this sense, we will use the FOM proposed in [32], given by (5). This FOM relates the gain–bandwidth product (GBP) to the noise figure and to the DC power consumption (see (6)).

(5) $$FOM=\frac{GBP}{\left(NF-1\right)\times P_{dc}}$$

where

(6) $$GPB={|S_{21}|}^{2}\times Bandwith$$

where ${|S_{21}|}^{2}$ and NF are the gain and small-signal noise factor, respectively, and $P_{dc}$ is the power consumption. In this research, the FOM of the proposed LNA is equal to 86 GHz/mW.

Regarding the double LNA structure, except for the non-existence of the CMRR, this is not a differential LNA MMIC. Therefore, it does not show rejection of the common mode. Additionally, the single-ended compression point value is slightly below the DLNA compression point shown in Section 4.3. The rest of the measured parameters of the dual single-ended LNA are of the order of the parameters obtained for the DLNA, taking into account the noise, the working frequency band, and the gain. If we make a comparison between both LNA MMICs, as shown in Table 2, it can be seen that the results obtained for the dual single-ended LNA and the DLNA are similar. However, it is worth noting that the compression point value of the DLNA is higher and that it has a good CMRR value, which is non-existent in most LNAs that are not of the differential design type. The shown values correspond to the expected values of the “on wafer” MMIC.

Finally, Table 3 provides a comparison of the DLNA design presented in this research with the state of the art.

## 5. Conclusions

In this paper, the design, simulation, and fabrication of two low-noise broadband amplifiers were presented. One was differential (DLNA), and another single-ended, using the 0.07 μm GaAs on metamorphic HEMT (mHEMT) technology from the European OMMIC foundry. In addition, the comparison between the differential amplifier and the other MMIC was carried out. The latter was based on a conventional design structure (single-ended). The results showed that the performance of the proposed DLNA is better than that of the other amplifiers.

Here, the DLNA and the single-ended LNA MMICs were measured at room temperature (290 K). The result was that a very good relationship was obtained between the simulations and the measurements carried out, both in input and output matching ($s_{11}$ and $s_{22}$, respectively) and in the gain obtained ($s_{21}$) at 290 K. Furthermore, the results showed a gain of 29 ± 2 dB over the entire bandwidth (1–16 GHz) at room temperature, with NF < 1.3 dB for the “on jig” measurements and NF < 1.0 dB in the case of DLNA for the “on wafer” measurements of the OMMIC foundry. Also, the equivalent noise temperature is shown alongside the possibility that the data can be extrapolated to cryogenic conditions, 25 K. In this last case, the authors think that the DLNA should be mounted on a support.

In addition to presenting high gain in the range of 1 to 18 GHz, with relatively low power consumption, the proposed DLNA also has CMRR > 40 dB. In this research, the stability study of the structure was also carried out, and, according to the classical stability metric, the μ factor achieved was greater than 1 in the entire frequency band that goes from 500 MHz to 40 GHz.

Finally, from our point of view, the research results demonstrate the great potential that the DLNA configuration has compared to mHEMT devices for applications in radio astronomy and microwave and millimeter wave sensing because the proposed DLNA MMIC has FOM = 144.5 GHz/mW, NF < 1.0 dB, and $P_{out1dB}$≥ 8.5 dBm.

## Figures and Tables

**Table 1:** D007IH key features.

$L_{g}$ (nm)	Thickness (μm)	$f_{T}$ (GHz)	$f_{\max}$ (GHz)	$g_{m\max}$ (mS/mm)	$I_{\mathrm{DS}\max}$ (mA/mm)	$V_{\mathrm{BD}}$ (V)
70	100	300	350	2500	600	3 (G-D)

Channel ${In}_{0.7}{Ga}_{0.3}As$ with *GaAs* substrate. where: $L_{g}$ is the gate width, Thickness is the “thickness of wafer”, $f_{T}$ is the cutoff frequency, $f_{max}$ is the maximum frequency at which the transistor can operate as an oscillator, $g_{m}$ is the transconductance, $I_{DS}$ is the drain current, and $V_{BD}$ is the gate-drain breakdown voltage.

**Table 2:** Characteristic parameters of the MMICs manufactured in this research.

Parameter	Dual Single-Ended LNA	DLNA
Gain	30.5 ± 2 dB	30.5 ± 2 dB
BW	1–16 GHz	1–16 GHz
ORL	<−14 dB	<−14 dB
$P_{out1dB}$	5.9 dBm	8.5 dBm
NF	1	1
*T*	90 K	90 K
Stability	OK	OK
FOM	86 GHz/mW	145.5 GHz/mW
CMRR	NA	≈40 dB

**Table 3:** Performance Comparison of the LNAs MMICs.

Parameter	DLNA	[2]	[22]	[24]	[42]	[73]
Process	70 nm GaAs	130 nm InP GaAs	NA Hybrid	130 nm CMOS	250 nm GaAs	130 nm CMOS
Gain (dB)	30.5 ± 2	13	16	16	<20	<13
BW (GHz)	1–16 GHz	0.5–13 GHz	0–1.2 GHz	1–6 GHz	1–10 GHz	7–15 GHz
$P_{out1dB}$ (dBm)	8.5 dBm	NR	NR	−8 dBm	10–14 dBm	NR
NF (dB)	1	<1	<1.6	4.7	<2.36	>3.1
FOM (GHz/mW)	145.5	≈52	≈0.2	2.1	≈4	1.07
CMRR (dB)	≈40	NA	27	NA	NA	NR
Chip Size (mm × mm)	1.5 × 2	2 × 0.75	22.4 × 16.6	0.4 × 0.6	2.7 × 1.7	2.1 × 1.185

Where: NR = Not Reported. NA = Not Applicable.

## Data Availability

Data are contained within the article.
